# A comprehensive summary of the ASEV-CzeSEV joint meeting on extracellular vesicles

**DOI:** 10.20517/evcna.2024.86

**Published:** 2024-12-21

**Authors:** Kristyna Turkova, Jan Balvan, Gabriela Ambrozova, Andrea Galisova, Martina Hyzdalova, Carla Tripisciano, Viktor Cerny, Irma Schabussova, Wolfgang Holnthoner, Vendula Pospichalova

**Affiliations:** ^1^Department of Biophysics of Immune System, Institute of Biophysics of the Czech Academy of Sciences, Brno 612 00, Czech Republic.; ^2^International Clinical Research Center, St. Anne’s University Hospital Brno, Brno 602 00, Czech Republic.; ^3^Department of Pathological Physiology, Faculty of Medicine, Masaryk University, Brno 625 00, Czech Republic.; ^4^Department of Radiodiagnostic and Interventional Radiology, Institute for Clinical and Experimental Medicine, Prague 140 21, Czech Republic.; ^5^Department of Pharmacology and Toxicology, Veterinary Research Institute, Brno 621 00, Czech Republic.; ^6^Department of Medicine I, Division of Hematology and Hemostaseology, Medical University of Vienna, Vienna 1090, Austria.; ^7^Institute of Specific Prophylaxis and Tropical Medicine, Centre for Pathophysiology, Infectiology and Immunology, Medical University of Vienna, Vienna 1091, Austria.; ^8^Ludwig Boltzmann Institute for Traumatology, the Research Centre in Cooperation with AUVA, Vienna 1200, Austria.; ^9^Austrian Cluster for Tissue Regeneration, Vienna 1200, Austria.; ^10^Department of Experimental Biology, Faculty of Science, Masaryk University, Brno 625 00, Czech Republic.

**Keywords:** Extracellular vesicles (EVs), EV biomarkers, cancer diagnostics, EV therapeutics, tissue regeneration, EV isolation techniques, cellular and interspecies communication

## Abstract

This report summarizes the ASEV-CzeSEV Joint Meeting on Extracellular Vesicles (EVs), held at the Medical University of Vienna in September 2024. The conference focused on introducing and expanding EV research and infrastructure within the Czech Republic and Austria, highlighting areas for collaboration. Key sessions featured research on EV-based diagnostics, tissue regeneration, interspecies communication and therapeutic applications, with an emphasis on shared resources and cross-border partnerships. The program included oral and poster presentations on EV engineering, new isolation techniques, and potential clinical applications, as well as industry updates on the latest EV technologies. The meeting concluded with awards for outstanding presentations reflecting the quality of work presented. Following the conference, a dedicated workshop was held on flow cytometry analysis of EVs, allowing participants to deepen their technical expertise in EV characterization. This report captures the main discussions, findings, and collaborative opportunities explored at the ASEV-CzeSEV meeting, signaling strong regional support for advancing EV research.

**Highlight:** This comprehensive report offers a deep dive into the sessions and highlights of the ASEV-CzeSEV Joint Meeting, held in Vienna in September 2024. The report covers all key aspects, including oral and poster presentations, and awards.

## INTRODUCTION

**Figure 1:**
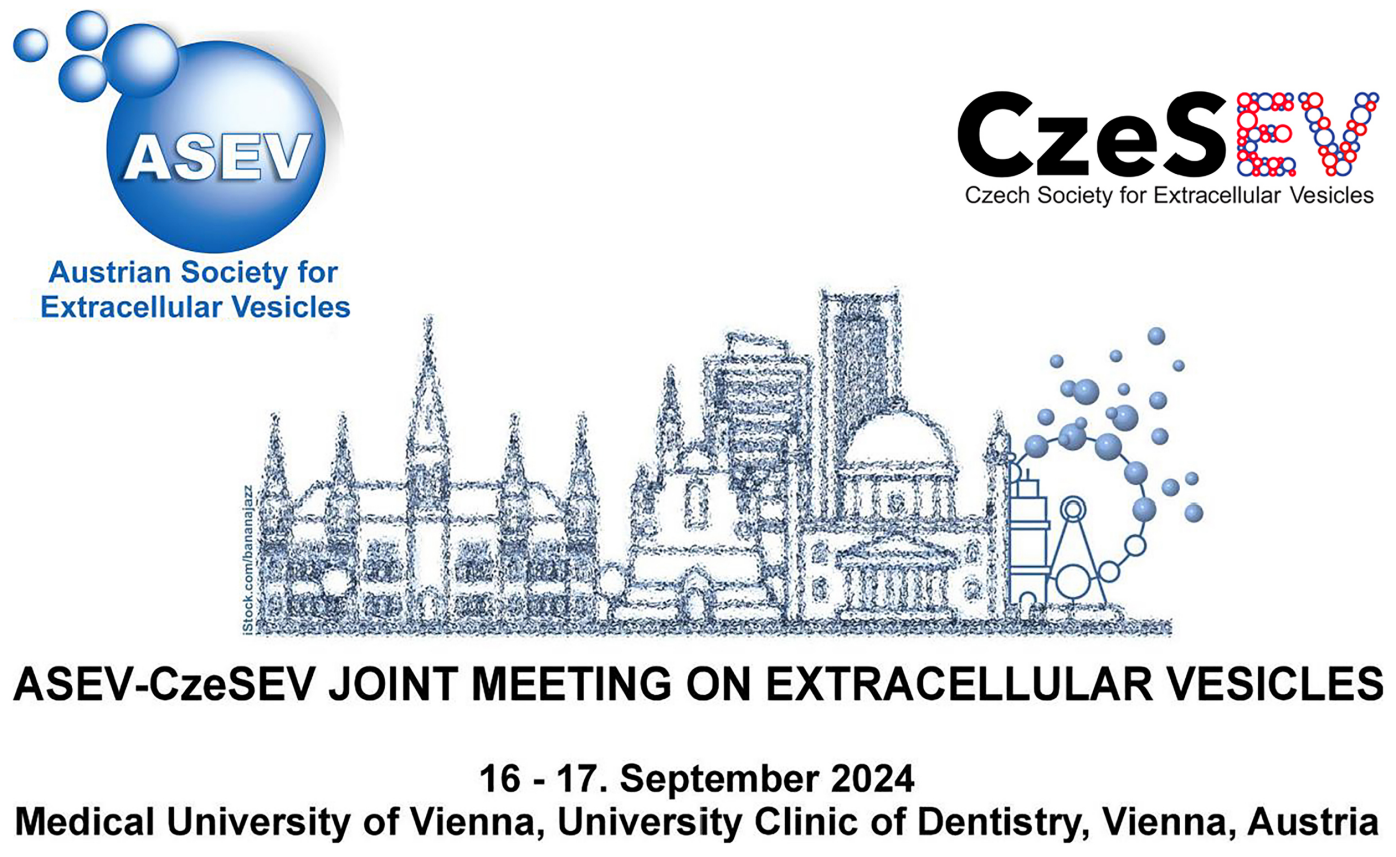
Logo of the ASEV-CzeSEV2024 conference. Designed by Carla Tripisciano.

Extracellular vesicles (EVs) have gained significant scientific attention over the past decade, from understanding their biology to applying them in diagnostics and therapeutics. This joint meeting of the Austrian Society for Extracellular Vesicles (ASEV, https://www.asev.at) and the newly founded Czech Society for Extracellular Vesicles (CzeSEV, https://www.sci.muni.cz/czesev) took place on September 16-17, 2024, at the modern facilities of the University Clinic of Dentistry, Medical University of Vienna [Figure 1].

**Figure 2:**
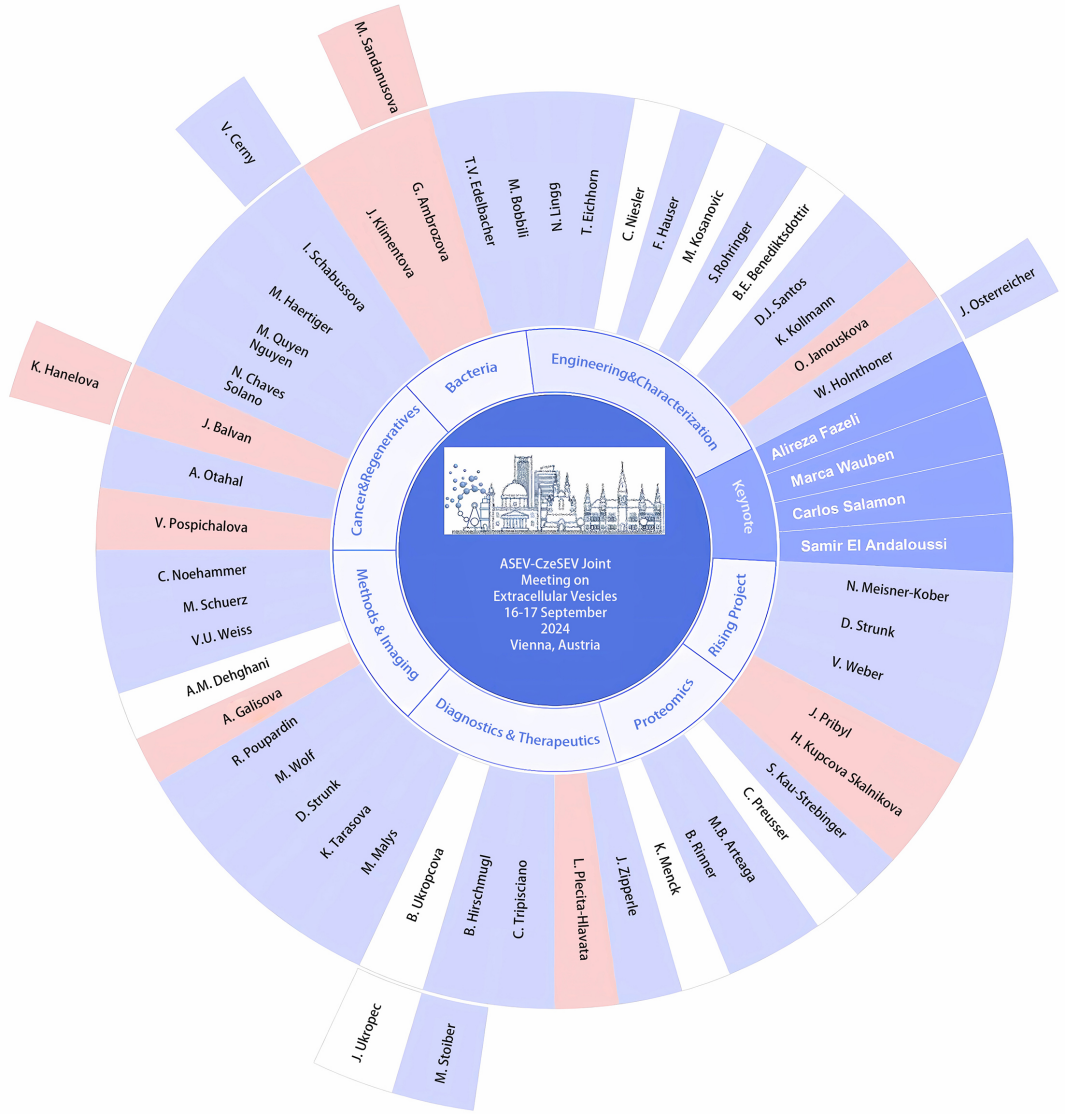
Mind map of ASEV-CzeSEV2024. Designed by Kristyna Turkova.

This two-day event, hereafter abbreviated as ASEV-CzeSEV 2024 (https://www.asev.at/annualmeeting), showcased cutting-edge research in EV biology, purification technologies, and potential applications in diagnostics and therapeutics. It brought together leading researchers, young scientists, and industry professionals from over 10 countries [Figure 2].

**Figure 3:**
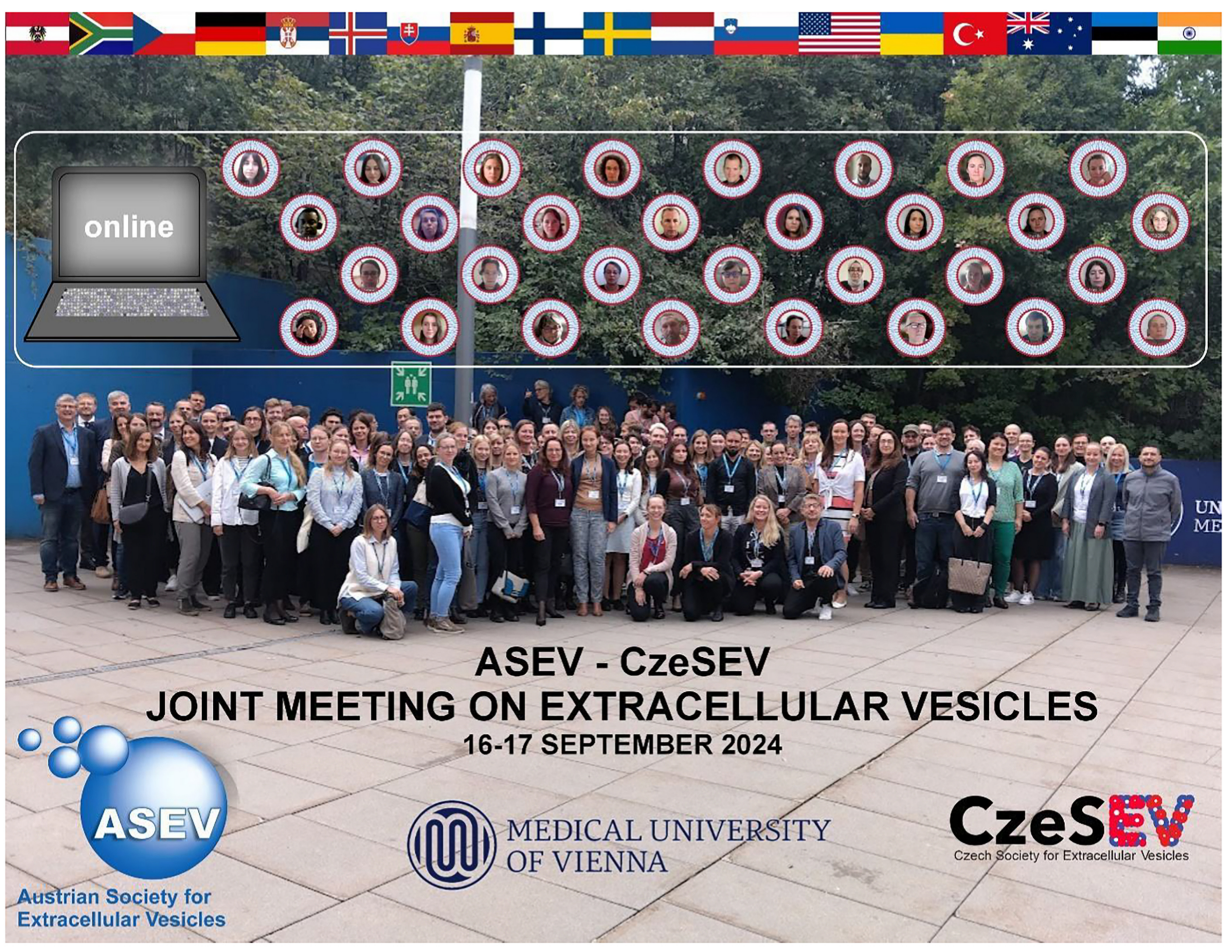
Participants of the ASEV-CzeSEV2024. Designed by Carla Tripisciano.

Due to the ongoing floods in both Austria and the Czech Republic, as well as other countries in Central Europe, the meeting unexpectedly had to be extended to a hybrid format. Nevertheless, over 100 participants attended in person, while more than 50 joined remotely via Zoom [Figure 3].

Continuing the tradition of collaboration with other European EV societies, the event featured four invited international keynote speakers, introduced nineteen research groups and included thirty presentations on original scientific work. Additionally, four “rising projects” (a summary of the MISEV2023 guidelines, an introduction of a newly established EV research institution, a newly founded EV doctoral school, and newly available infrastructure for EV research) from Austria and the Czech Republic were highlighted, alongside an extensive poster session and updates from the sponsoring companies, emphasizing the importance of cooperation in advancing EV research.

The day following the conference, a workshop focused on the flow cytometry (FC) of EVs was held at the Vienna One Vision Center, organized in collaboration with Beckman Coulter Life Sciences (BCLS).

## DAY 1 - MORNING SESSION

The opening session, chaired by Irma Schabussova *(*Austria, Medical University of Vienna) and Miriam Sandanusova (Czech Republic, Czech Academy of Sciences*)*, highlighted the diverse roles of EVs in cancer progression and regenerative medicine. Discussions highlighted their potential as both biomarkers and therapeutic agents, particularly in ovarian cancer, orthopedic therapies, and nerve regeneration.

The event was opened with the keynote lecture given by **Marca Wauben** *(*Netherlands, Utrecht University), who discussed the physiological effects of human milk-derived EVs on early immune development. Her lab’s research has demonstrated that milk EVs possess immune-regulatory properties and enhance the formation of the intestinal barrier, a critical tissue for newborn health. They showed that milk EVs induce migration, but not proliferation, of certain cell types. A key innovation from her lab is the development of high-resolution FC techniques, which enable a more detailed analysis of individual EVs, moving beyond bulk analysis to study specific vesicle subsets^[1]^. This technology promises breakthroughs in identifying EV-based biomarkers and enhancing our understanding of EV heterogeneity in health and disease. Marca’s research suggests a potential therapeutic application of EVs in areas such as neonatal health, immune modulation, and as carriers for drug delivery.

**Vendula Pospichalova** (Czech Republic, Masaryk University), President of CzeSEV, presented an introduction to her research group. Her lab focuses on EVs from ascites in ovarian cancer, providing critical insights into disease progression. Ovarian cancer, particularly high-grade serous carcinoma (HGSC) of the ovary, fallopian tube, and peritoneum, remains a major challenge in women’s health due to its elusive nature and resistance to treatment^[2]^. Dr. Pospichalova’s research aims to decode the molecular mechanisms driving disease progression, using ascites-derived EVs as potential biomarkers for diagnosis, screening, and prognosis in HGSC.

The group of **Alexander Otahal** (Austria, University for Continuing Education Krems) studies EVs in the context of orthopedic therapies, particularly for cartilage and bone regeneration. Their research includes the production, enrichment, and characterization of EVs derived not only from blood products but also from mesenchymal stem cells (MSCs) harvested from Hoffa’s fat pad and cultured in dynamic bioreactors. These EVs are being investigated for their potential application in tissue engineering, including their integration into 3D bioprinting techniques for cartilage repair and regeneration.

**Jan Balvan** (Czech Republic, Masaryk University), together with PhD student **Klara Hanelova**, presented their team’s research on the role of EVs in head and neck cancer, with a focus on their potential as biomarkers. Their work investigates changes in the protein and RNA content of phosphatidylserine-positive EVs derived from cancer-associated fibroblasts and cancer cells under both normal and stress conditions, such as lysosomal dysfunction and nutrient deprivation. Additionally, Jan’s team investigated how these EVs influence recipient cell behavior, including migration and sensitivity to cell death, highlighting the dynamic interaction between cancer cells and cancer-associated fibroblasts.

Subsequent presentations of original research highlighted the regenerative potential of EVs. The talk of **Nefertiti Chaves Solano** (Austria, Ludwig Boltzmann Institute for Traumatology) presented the therapeutic potential of the human amniotic membrane (hAM) for tissue regeneration. This study identified pro-regenerative miRNAs in hAM’s secretome, analyzing EV-contained and protein-bound miRNA profiles and their distribution across hAM subregions. Results revealed miRNA clusters associated with tissue regeneration pathways, suggesting potential for regenerative applications, including osteoblast regeneration and wound healing.

In the following presentation, **Mai Quyen Nguyen** (Austria, Ludwig Boltzmann Institute for Traumatology) discussed how adipose-derived stem cell EVs can enhance peripheral nerve regeneration. The study demonstrated the development of laminin-binding EVs derived from adipose stem cells, engineered to target injured peripheral nerves selectively. Promising *in vitro* results showed that these EVs enhanced Schwann cells (SCs) uptake and increased expression of regenerative genes, aiming to improve recovery in peripheral nerve injury models.

**Maximilian Haertinger** (Austria, Medical University of Vienna) presented a study exploring how small EVs from adipose stromal cells (ASCs) impact SCs in injury response. Results showed ASC-EVs enhanced SCs’ immunomodulation, myelin clearance, and remyelination, with promising implications for nerve regeneration.

**Nicole Meisner-Kober** (Austria, Paris Lodron University of Salzburg) introduced the newly founded Ludwig Boltzmann Institute in Austria, which will explore novel strategies for utilizing EVs in translational settings to target diseases at a personalized medicine level.

## DAY 1 - AFTERNOON SESSION 1

After the General Assembly of ASEV and lunch, the conference program continued with the afternoon session, chaired by Andrea Galisova (Czech Republic, Institute for Clinical and Experimental Medicine) and Adrian Klepe (Austria, Austrian Institute of Technology), highlighted the therapeutic potential of EVs from probiotics and pathogens, showing their relevance in immune modulation, allergy prevention, and vaccine development.

**Carlos Salomon** (Australia, University of Queensland) presented a compelling keynote lecture on translational biomarkers for ovarian cancer from circulating EVs. As a leading figure in gynecologic cancer research, Carlos has dedicated over a decade to improving diagnostic methods for ovarian cancer. The focal point of his talk was the Ovarian Cancer Research Foundation-7 (OCRF-7) EV Ovarian Cancer Study, a project that examines blood-based biomarkers as early indicators of ovarian cancer. Carlos highlighted significant findings in this study, notably a new biomarker panel based on seven specific proteins in blood samples. This panel demonstrated a classification efficiency exceeding 90% in detecting early-stage ovarian cancer, representing a significant advancement over the traditional CA125 test, which has long been used but lacks similar precision for early detection. Carlos further emphasized the transformative potential of this new approach, with the biomarker panel offering a much-needed enhancement in early ovarian cancer diagnosis, ultimately paving the way for improved patient outcomes. His team’s work reflects a major stride in translational medicine, as they continue to bridge the gap between laboratory discoveries and real-world clinical applications.

**Gabriela Ambrozova** (Czech Republic, Czech Academy of Sciences) introduced her research group, which focuses on studying EVs in inflammation-related diseases. They investigate the impact of both bacterial and host-derived EVs, particularly how bacterial EVs from microbiota influence immune cells and modulate the production of host EVs. A significant aspect of their work is the immunomodulatory potential of EVs, especially those derived from beneficial bacteria like *Lacticaseibacillus*.

This was the main theme of PhD student **Miriam Sandanusova’s** follow-up presentation, which delved into her recent publication^[3]^ on the immunomodulatory effects of membrane vesicles (MVs) from *Lacticaseibacillus rhamnosus,* analyzed across different bacterial growth phases.

**Irma Schabussova** (Austria, Medical University of Vienna) then discussed the role of EVs derived from probiotic bacteria in allergy prevention, emphasizing their potential for therapeutic use in mucosal applications. Her research focuses on isolating and characterizing EVs from probiotic bacteria to develop mucosal therapies for allergic diseases. Recent findings suggest that EVs from probiotic *E. coli* O83 (EcO83-EVs) can enhance immune responses in human nasal epithelial cells and reduce allergic airway inflammation in mice, highlighting their potential as a safer alternative to live probiotics in therapeutic applications^[4]^.

Building on this, **Viktor Cerny** presented work from Schabussova’s group on engineering EcO83-EVs, decorated with the SpyCatcher-SpyTag system, designed to modulate immune responses further, which could advance allergen-specific tolerance induction.

**Jana Klimentova** (Czech Republic, University of Defense) shared insights into *Francisella tularensis* EVs and their potential use in vaccine development. Her study investigates the role of outer MVs and nanotubes produced by *Francisella tularensis* in host-pathogen interactions, particularly how they facilitate bacterial entry into macrophages while eliciting a strong pro-inflammatory response. Additionally, her research explores these vesicles for vaccine development against tularemia, focusing on their protective effects and the search for mutant strains with enhanced vesicle production.

Finally, **Tanja V. Edelbacher** (Austria, University of Veterinary Medicine) addressed the issue of mechanisms of EV secretion from the Gram-positive bacterium *Bacillus cereus*. Their study examined how culture conditions influence EV secretion and composition, revealing that media selection affects EV lipid, protein, and RNA profiles. Findings suggest that the bacteria’s metabolic state and lipid metabolism impact EV diversity, underscoring the importance of culture conditions for understanding EV-mediated infection mechanisms.

## DAY 1 - AFTERNOON SESSION 3

The afternoon session led by Klara Hanelova (Czech Republic, Masaryk University) and Alexander Otahal (Austria, University for Continuing Education Krems) focused on the therapeutic potential of EVs and their roles during the healing and treatment processes.

**Dirk Strunk** (Austria, Paracelsus Medical University) had a quite challenging task, since the time for the presentation of “rising projects” was just 5 min. Within this time, he managed to summarize the key points in the MISEV2023 guidelines, which are important for all EV researchers.

**Madhusudhan Bobbili** (Austria, Ludwig Boltzmann Institute for Traumatology) presented a targeted approach to developing EVs for drug delivery. The research addresses challenges such as EV heterogeneity, contaminants, and limited understanding of cargo release. Madhu’s team developed a library of CD81 variants to target proteins such as laminin, EGFR, and Her2 in overexpressing cells, enhancing EV specificity for EGFR and Her2. They engineered bi-specific EVs for better targeting and internalization and used VSV-G fusion protein to ensure endo-lysosomal escape, protecting the cargo. Advanced purification techniques, including the use of CLIP-tag and PreScission protease, were used to achieve high-purity EVs by removing extracellular chromatin and other contaminants^[5]^.

**Nico Lingg** (Austria, University of Natural Resources and Life Sciences, BOKU University in Vienna) demonstrated the use of non-woven fibers and tangential flow filtration (TFF) for the purification step following the isolation of EVs, which enabled the efficient removal of host cell DNA. This may lead to the production of pure and safer EVs for administration during late-phase clinical trials of EV-based drugs.

Another “rising project” in Austria is exemplified by a new PhD program “EVision”, involving the University for Continuing Education in Krems, the University of Applied Sciences in Krems, and the Medical University of Vienna, offering four positions for PhD students in the field of EV research as was presented by **Viktoria Weber** (Austria, University for Continuing Education Krems).

An interesting talk regarding the role of EVs released during shock wave therapy in the angiogenesis of ischemic tissue was presented by **Tanja Eichhorn** (Austria, University for Continuing Education Krems). Using human umbilical vein endothelial cells (HUVECs) as an experimental model, they demonstrated an increase in the production of EVs after exposure of cells to shock wave therapy, as well as the involvement of the Hippo pathway in these processes. In addition, a clinical study showing the effect of shock wave therapy in ischemic cardiomyopathy is also underway.

The next speaker was **Carola Niesler** (South Africa, University of KwaZulu-Natal in Pietermaritzburg), who talked about small EVs as possible carriers of the pro-regenerative effects of human umbilical cord blood serum (hUCBS). In their study, they compared the protein cargo of whole serum with that of small EVs isolated from hUCBS. As expected, the hUCBS was rich in proteins, many of which were also found in the small EVs. Moreover, the levels of some of them (e.g., β-NGF, IL-15 and TNF-α) were significantly higher in the EVs compared with the hUCBS, suggesting a possible role of the EVs in healing and regeneration processes.

**Fabian Hauser** (Austria, University of Applied Sciences Upper Austria) presented his research on visualizing actin in EVs and its redistribution during cellular uptake at the single-molecule level. Using a genetically modified HEK293T cell line and fluorescence microscopy, they detected actin in EVs and analyzed its spatial distribution. The findings provide insights into subpopulations of EVs carrying actin and their potential physiological roles in intercellular communication.

The next presentation given by **Maja kosanović** (Serbia, University of Belgrade) showed the potential of EVs from *Trichinella spiralis* muscle larvae (TsEVs) to alleviate ovalbumin (OVA)-induced allergy in a murine model. Treatment with TsEVs significantly reduced OVA-specific IgE levels, decreased eosinophil counts, and modulated immune cell populations in the lungs. Additionally, TsEVs increased regulatory T cells and IL-10 production while reducing Th2 cytokines, suggesting their potential as immunomodulatory agents for treating respiratory allergies.

Finally, **Sabrina Rohringer** (Austria, Medical University of Vienna) presented a study investigating the role of EVs from perivascular adipose tissue in the healing of small-diameter vascular grafts. The results showed that EVs from expanded polytetrafluoroethylene grafts had reduced angiogenic potential and a more pro-inflammatory profile as compared to autologous vessels and thermoplastic polyurethne grafts. These findings suggest that impaired EV-mediated cell communication may contribute to the limited healing and endothelialization of expanded polytetrafluoroethylene grafts.

## POSTER PRESENTATIONS

The first day of ASEVCzeSEV2024 concluded with a poster session. Unfortunately, due to unforeseen circumstances, about one-third of the posters were not presented, as many participants could not attend in person because of the floods. Despite this, the featured research spanned a wide array of topics, showcasing the potential of EVs in diagnostics and therapeutic applications, and exploring fundamental biological mechanisms. This session allowed scientists to present their work and provided ample networking opportunities with other researchers and industry leaders. Here are some of the most captivating highlights from the poster presentations:

### 1. EV uptake and release in a human *in vitro* blood-brain barrier co-culture model under ischemic conditions

**Authors**: Adrian Klepe, Ana Spilak, Andreas Brachner, Christa Noehammer, Winfried Neuhaus

Austrian Institute of Technology, Austria.

**Summary**: This poster explores the dynamics of EVs in the context of blood-brain barrier integrity under ischemic conditions. The study uses a human *in vitro* blood-brain barrier co-culture model and examines how ischemia affects EV uptake and release, providing insights into potential EV-based therapies for stroke and other neurological conditions.

### 2. NaTaLi: Nanobody-Tag Ligand click strategy for targeted multicolor EVs

**Authors**: Andrea Galisova^1^, Ester Merunkova^1^, Jiri Zahradnik^2^, Daniel Jirak^1^

^1^Institute for Clinical and Experimental Medicine, Czech Republic. ^2^Charles University, Czech Republic.

**Summary**: This research presents the “NaTaLi” system, which utilizes nanobodies displayed on the surface of EVs to bind specifically to tagged ligands. This highly versatile system has applications in multiplexed cancer biomarker detection and targeted drug delivery, potentially advancing cancer diagnostics and therapy.

### 3. Extracellular vesicles from dental pulp and bone marrow mesenchymal stromal cells: a comparative analysis

**Authors**: Maryna Shamshur^1,2^, Nadiia Petryk^1^, Alona Zlatska^1,2^, Inna Godriienko^1,3^

^1^Medical Company “Good Cells”, Ukraine. ^2^National Academy of Medical Sciences of Ukraine, Ukraine. ^3^R.E. Kavetsky Institute of Experimental Pathology, Ukraine.

**Summary**: This comparative study examines EVs from dental pulp and bone marrow MSCs, highlighting differences in their content and potential uses in regenerative medicine. EVs from dental pulp were found to contain higher levels of neuroregenerative factors, while bone marrow-derived EVs had stronger angiogenic potential.

### 4. Extracellular vesicles released from macrophages infected with intracellular pathogenic bacterium *Francisella tularensis*

**Authors**: Ivona Pavkova^1^, Jana Klimentova^1^, Jitka Zakova^1^, Jaroslav Hanus^2^, Erik Vlcak^3^, Michaela Hudakova^4^

^1^University of Defence, Czech Republic. ^2^University of Chemistry and Technology, Czech Republic. ^3^Czech Academy of Sciences, Czech Republic. ^4^Charles University, Czech Republic.

**Summary**: This poster investigates how *Francisella tularensis* manipulates host cells through EV release. The study shows that the bacteria trigger the release of specific EVs from infected macrophages, which play a crucial role in modulating immune responses. This suggests potential applications of bacterial EVs in understanding host-pathogen interactions and developing new therapeutic approaches.

### 5. Analysis of exosomes isolated from tumor and non-tumor tissue samples

**Authors**: Nicol Strakova^1^, Monika Levkova^2,3^, Zdenek Kolar^2,3^ Jan Bouchal^2,3^, Samuel Genzor^3^, Karolina Strakova^2^, Simona Strapacova^1^, Jan Kotoucek^1^, Pavel Kulich^1^, Ondrej Kovac^1^ Miroslav Machala^1^, Josef Masek^1^, Martina Hyzdalova^1^

^1^Veterinary Research Institute, Czech Republic. ^2^Palacky University, Czech Republic. ^3^University Hospital Olomouc, Czech Republic.

**Summary**: This poster presents a comparative analysis of exosomes isolated from both tumor and non-tumor tissues. The findings provide key insights into the differences in exosome composition between these two sources, with potential implications for cancer diagnostics and targeted therapy.

### 6. Lipidomics of EVs in response to aerobic exercise

**Authors**: Dominika Olesova^1^, Ales Kvasnicka^2,3^, Martin Schon^4^, Igor Straka^5^, Zuzana Kosutzka^5^, Peter Valkovic^5^, David Friedecky^2,3^, Jozef Ukropec^4^, Barbara Ukropcova^4^

^1^Slovak Academy of Sciences, Slovakia. ^2^Comenius University, Slovakia. ^3^Comenius University and University Hospital of Bratislava, Slovakia. ^4^Biomedical Research Center, Slovak Academy of Sciences, Slovakia. ^5^Medical University of Vienna, Austria.

**Summary**: This study explores the changes in lipid profiles of EVs in response to acute aerobic exercise. The findings suggest that exercise-induced lipidomic changes in EVs could have implications for understanding exercise-related adaptations in human health.

### 7. Host-adapted molecular signatures in *Toxoplasma gondii* EVs

**Authors**: Teresa Cruz Bustos, Anna Sophia Feix, Anja Joachim

University of Veterinary Medicine Vienna, Austria.

**Summary**: The research delves into how *Toxoplasma gondii* utilizes EVs to manipulate host cell processes. The study focused on analyzing proteomic and lipidomic profiles from EVs released during infections, which could pave the way for new treatments targeting this parasite.

### 8. Protective effects of MSC-derived EVs on the blood-brain barrier

**Authors**: Vojtech Sprincl^1,2^, Kristyna Sintakova^1,2^, Nataliya Romanyuk^1^

^1^Czech Academy of Sciences, Czech Republic. ^2^Charles University, Czech Republic.

**Summary**: This poster investigates the potential of MSC-derived EVs in protecting the blood-brain barrier. The research could lead to novel therapeutic approaches for neurological disorders involving blood-brain barrier dysfunction.

### 9. Scalable isolation and characterization of milk-derived extracellular vesicles for therapeutic applications

**Authors**: Atefeh Ebrahimian^1^, Lukas Herzog^1^, Mona Kandel^1^, Mona Schalk^1,^, Melanie Pfitzner^1,2^, Veronika Wimmer^1,3^, Laurentius Orsolic^1^, Michael Maurer^1^, Christiane Gebhard^1^, Harald Kühnel^1^

^1^FH Campus Wien, Austria. ^2^Biotech Campus Tulln, Austria. ^3^University of Applied Sciences Krems, Austria.

**Summary:** This research focuses on the isolation and characterization of milk-derived EVs for potential therapeutic use. The team developed a scalable method to purify EVs from milk, aiming to utilize their bioactive properties.

### 10. Endothelial extracellular vesicles in vascular dysfunction associated with chronic kidney disease

**Authors**: Andrea Figuer^1,2^, Fatima Milhano Santos^3^, Sergio Ciordia^3^, Gemma Valera^4,5^, Beatriz Martín-Jouve^5^, Juan Pablo Hernandez^3^, Guillermo Bodega^6^, Rafael Ramirez^1,2^, Julia Carracedo^4,5^, Matilde Alique^1,2^

^1^Universidad de Alcalá, Spain. ^2^Instituto Ramón y Cajal de Investigación Sanitaria (IRYCIS), Spain. ^3^Centro Nacional de Biotecnología, Campus de Cantoblanco, Spain. ^4^Universidad Complutense de Madrid, Spain. ^5^Instituto de Investigación Sanitaria Hospital, Spain. ^6^Universidad de Alcalá, Spain.

**Summary:** This study explores how EVs secreted by endothelial cells contribute to vascular dysfunction in patients with chronic kidney disease. Their findings suggest that EVs may play a role in the progression of vascular complications in such conditions.

### 11. The parasite *Schistosoma mansoni* triggers the host cells to release extracellular vesicles with immunomodulatory properties

**Authors:** Michael Thaler^1^, Lukas Neuninger^1^, Magdalena Wysmołek^1^, Agnieszka Razim^1^, Anna Schmid^1^, Jan Dvorak^2^, Martin Horn^3^, Adrian Leontovyc^3^, Michael Mares^1^, Christoph Nagl^1^, Muhammed Faruk Saglam^1^, Karin Hoffmann-Sommergruber^1^, Viktor Cerny^1^, Aleksandra Inic-Kanada^1^, Ursula Wiedermann-Schmidt^1^, Irma Schabussova^1^

^1^Medical University of Vienna, Austria. ^2^Czech University of Life Sciences Prague, Czech Republic. ^3^Czech Academy of Sciences, Czech Republic.

**Summary:** *Schistosoma mansoni* uses proteases like Cathepsin B1 (SmCB1) and SmCL3 to invade the host through the skin and migrate through the lungs. These proteases influence the host’s immune response and induce the release of EVs in cell lines. EVs play a role in intercellular communication, and in parasitic infections, they can carry immunomodulatory signals.

### 12. Proteomic profiling reveals growth-dependent protein cargo alterations in *Lacticaseibacillus rhamnosus* membrane vesicles

**Authors**: Kristyna Turkova^1^, Martin Sindelar^2^, Miriam Sandanusova^1^, Eva Pechackova^1^, Lukas Kubala^1,2^, Gabriela Ambrozova^1^

^1^Czech Academy of Sciences, Czech Republic. ^2^Masaryk University, Czech Republic.

**Summary:** In this study, MVs were isolated from *L. rhamnosus* at early exponential, late exponential, and late stationary phases, and then analyzed using cryo-EM, NTA, and proteomic techniques. While the overall protein families and subcellular localizations remained consistent, significant differences were observed in the intensities of individual proteins, especially those involved in genetic information processing and carbohydrate metabolism. These findings suggest that the timing of MV harvest is crucial for studies focusing on MV function and potential applications.

## DAY 2 - MORNING SESSION 1

The second day of ASEVCzeSEV2024 was opened by a morning session, chaired by Dirk Strunk (Austria, Paracelsus Medical University, online) and Kristyna Turkova (Czech Republic, Czech Academy of Sciences), and highlighted cutting-edge advances in EV engineering, showcasing novel strategies for targeted cancer therapy, spinal cord injury (SCI) repair, acute myeloid leukemia (AML) treatment, and vascular and lymphatic applications, while also addressing diverse EV sources and innovative isolation methods.

The first talk was given by a keynote speaker **Samir El Andaloussi** (Sweden, Karolinska Institute). Samir’s presentation focused on the potential of EVs as versatile tools for drug delivery and cell engineering. He highlighted EVs’ key advantages, such as safety, cargo protection, and the ability to cross biological barriers, while acknowledging challenges like fast blood clearance, poor luminal loading, and limited endosomal escape. To overcome these issues, Samir proposed various engineering strategies to enhance EV efficacy.

Samir provided several examples of the application of genetic engineering for the creation of EV-based therapeutic vehicles. One of the examples was a platform for efficient RNA delivery using a fusion of an RNA-binding domain with CD63 in EV producer cells. Combining this with a fusogenic endosomal escape moiety (VSV-G) enabled functional mRNA delivery at lower doses compared to synthetic lipid nanoparticles.

Moreover, he presented innovative work on engineering EVs with the interleukin-6 signal transducer, which acts as a decoy receptor for inflammatory cytokines like TNF-α and IL-6. Modified EVs demonstrated superior performance compared to other agents in mouse models of systemic inflammation, neuroinflammation, and intestinal inflammation, suggesting a promising new strategy for treating inflammatory diseases. Samir concluded his talk by discussing antibody-decorated EVs for targeted cancer therapy. By adding an Fc-binding domain, EVs could be customized to target cancer cells expressing HER2 and PD-L1, resulting in significant tumor reduction in melanoma models. This modular system could be adapted for a variety of antibody-based cancer therapies.

**Berglind E. Benediktsdottir** (Iceland, University of Iceland) presented research on engineering EVs for targeted triple-negative breast cancer therapy. By genetically modifying HEK293 cells to express proteins that bind to EGFR receptors, her team developed EVs specifically targeting triple-negative breast cancer. They isolated these EVs using an immunoaffinity capture method, characterized them via western blot, and confirmed their uptake by cancer cells through confocal microscopy. The EVs were successfully loaded with doxorubicin using an ammonium gradient. Berglind introduced that her team is now exploring CRISPR to boost their therapeutic potential, aiming to translate their research into clinical practice.

**Diogo J. Santos** (Austria, Ludwig Boltzmann Institute for Traumatology) presented his work on engineering EVs from MSCs for SCI treatment. His research focuses on enhancing EV targeting while preserving their regenerative properties. Using genetically modified HEK293 cells, Diogo produced EVs expressing proteins associated with SCI, such as MAG (myelin oligodendrocyte glycoprotein, protein present in myelin sheaths that becomes disrupted during the acute phase of SCI) and NCAN (Neurocan, a chondroitin sulfate proteoglycan that is upregulated at the glial scar in the subacute to chronic phases of SCI). MAG/NCAN fused with CD81 mutants were selected from a yeast display library. These engineered EVs are designed to home to injury sites, bypassing capture by organs like the liver and spleen. The next steps involve assessing their targeting and therapeutic potential both *in vitro* and *in vivo*.

**Karoline Kollmann** (Austria, Veterinary Medicine University in Vienna) presented her research about the role of EV trafficking in AML. Karoline showed that cyclin-dependent kinase 6 (CDK6) plays an important role in AML. Using murine and human *in vitro* models, along with transcriptomic and proteomic analysis, Karoline found that CDK6 inhibition increased gene expression related to vesicles and lysosomes, enhancing EV release. The proteomic analysis also identified CDK6 interaction with vesicle proteins. Combined targeting of CDK6 and BRD4 boosted EV release and promoted cell death, revealing CDK6’s role in vesicle regulation and intercellular communication in AML.

**Olga Janouskova** (Czech Republic, Jan Evangelista Purkyne University in Usti nad Labem) presented her research group focused on the production and application of vesicles isolated from different sources such as human cancer cell lines, primary cell lines, human blood, plant and Spanish slug (Arion Vulgaris). The group implements EVs from tumor tissue and blood as biomarkers for glioblastoma and studies their role in tumor progression and immunity. They study improvements of EV properties by polymers, such as stabilization with polyoxazolines. Olga’s team also explores plant-derived EVs in regenerative medicine, optimizing their production and therapeutic loading with various therapeutic compounds (siRNA, doxorubicin) for therapy or vaccination purposes. The group also works on the development of microfluidic chips for EV research.

The next speaker of the session was **Wolfgang Holnthoner** (Austria, Ludwig-Boltzmann Institute for Traumatology), the current President of ASEV. Wolfgang started his talk by highlighting the significance of endothelial cells, which make up about one kilogram of the adult human body. He explained that if these cells were lined up, they would circle the earth twice. As the key components of vascular structures, they play a vital role in delivering oxygen and nutrients to tissues and draining interstitial fluid via lymphatic vessels. Wolfgang then shifted focus to his decade-long research on EVs. He presented equipment that his team utilizes for EV enrichment, such as ultracentrifugation (UC), TFF, and size exclusion chromatography (SEC), and for EV analysis, which includes nanoparticle tracking analysis (NTA), FC western blotting, and thromboelastometry. His current research examines EVs derived from endothelial cell cultures and human plasma, aiming to discover EV biomarkers for vascular diseases such as lymphedema.

**Johannes Oesterreicher** (Austria, Ludwig Boltzmann Institute for Traumatology), PhD student of Wolfgang Holnthoner**,** presented his work on engineered EVs for activation of lymphangiogenesis, which is crucial for tissue repair after injuries and diseases. Johannes’ research examines vascular endothelial growth factor C (VEGF-C), a key driver of lymphatic vessel formation, and its interaction with VEGFR-3. To improve VEGF-C’s bioavailability and targeting, the project links it to CD81 EVs. Stable cell lines expressing CD81-VEGF-C fusion proteins were created to activate receptors and target tissues. Early tests showed differences in receptor activation and confirmed pro-lymphangiogenic function, laying the groundwork for future *in vivo* trials to promote lymphangiogenesis.

## DAY 2 - MORNING SESSION 2

This session, chaired by Carla Tripisciano (Austria, Medical University of Vienna) and Martin Smolko (Czech Republic, Masaryk University) focused on the significant advancements in EV research and cell-based therapies, emphasizing the importance of rigorous characterization and standardization in the rapidly evolving field. Key topics included the development of automated analytical tools and bioinformatics workflows to enhance the reliability and comparability of EV studies, as well as novel platforms for efficient data reporting and literature navigation.

**Dirk Strunk** (Austria, Paracelsus Medical University) introduced the Cell Therapy Institute, where he serves as Director. Their research focuses on advancing cell- and EV-based therapies in regenerative medicine by developing scalable manufacturing and isolation platforms for human induced pluripotent stem cell-derived EVs and stromal cells, using both 2D and 3D production methods. Key findings include the influence of oxygen and heparin on EV function, the discovery of EV protein corona effects, and evidence that EV-rich platelet lysates support bone and vascular regeneration, emphasizing the potential for clinical translation.

**Martin Wolf** (Austria, Paracelsus Medical University) reported about another project from the Cell Therapy Institute. To address the overwhelming volume of EV-related research, their study developed a bioinformatics workflow for a comprehensive meta-analysis, uncovering key associations between EV sources, isolation methods, cargo, and functions. Findings revealed distinct clustering patterns, notably separating EV cargo studies from functional analyses, providing a “literature map” that offers valuable methodological insights for targeted EV applications.

**Rodolphe Poupardin** (Austria, Paracelsus Medical University) informed us about the EV-Zone platform (www.ev-zone.org), which provides tools to support standardized reporting and efficient literature searches in EV research. The EV-Checklist tool helps researchers quickly prepare consistent EV research reports for manuscripts, while the EV-PMC Search tool offers powerful search options for exploring over 22,000 open-access EV publications. Together, these tools enhance transparency, accessibility, and research efficiency in the EV field.

**Andrea Galisova** (Czech Republic, Institute for Clinical and Experimental Medicine) presented her research that leverages magnetic resonance imaging (MRI)-based molecular imaging to track engineered EVs that are targeted to specific tissues and loaded with contrast agents, enhancing their diagnostic and therapeutic capabilities. By combining protein engineering with advanced MRI techniques, the team aims to develop EV-based systems for precise, non-invasive drug delivery and monitoring, with applications like targeted cancer therapy.

**Aslan Mehdi Dehghani** (United States) introduced an analytical toolbox consisting of multiple orthogonal techniques for the analysis of EV samples. The analytical toolbox consists of NTA, FC, and analytical high-performance liquid chromatography. Importantly, the assays are established according to MISEV2023 and MIFlowCyt-EV guidelines.

**Victor U. Weiss** (Austria, Vienna University of Technology) presented a nano electrospray gas-phase electrophoretic mobility molecular analyser (nES GEMMA) system, which separates single-charged particles based on electrophoretic mobility diameter, enabling precise characterization of nanoparticles. This study adapted nES GEMMA from analyzing liposomes to EVs from human blood, achieving size distribution and particle concentration measurements. Findings highlight the system’s capability for EV analysis, essential for their potential application as pharmaceutical delivery vehicles.

**Jan Pribyl** (Czech Republic, Central European Institute for Technology, Masaryk University) introduced laboratories within CzeSEV that provide comprehensive analyses of EVs, focusing on their structure, mechanical properties, and chemical composition, as well as their interactions with tissues. The Core Facility Nanobiotechnology at Central European Institute for Technology, Masaryk University, specializes in nanoscale to microscale characterizations using advanced techniques such as biological atomic force microscopy, nanoindentation, and Raman microscopy, offering insights into elasticity, chemical composition, and cellular electrophysiology under physiological conditions.

**Melanie Schuerz** (Austria, Paris Lodron University of Salzburg) introduced EVAnalyzer, an ImageJ plugin for automated, quantitative single vesicle analysis, which has gained significant attraction within the EV community, boasting over 2,500 downloads. Building on this success, Melanie and colleagues created ImageC, an open-source image processing software in C++, leading to the release of EVAnalyzer2, which offers enhanced capabilities for tracking EVs in complex *in vitro* and *in vivo* models, supports large image sizes, and incorporates AI-driven object detection for improved vesicle quantification in extensive histological sections.

**Christa Noehammer** (Austria, Austrian Institute of Technology) highlighted multi-omics strategies at the forefront of personalized medicine and biomarker development, emphasizing that complex diseases cannot be fully understood through isolated measurements alone; rather, the interactions among genes, transcripts, proteins, and environmental factors play a critical role. By showcasing examples from their studies on immune-mediated and cardiovascular diseases, the team highlights the potential of multi-omics and EV-derived biomarker profiling in creating multi-modal diagnostic biomarkers and deepening the understanding of disease pathology.

## DAY2 - AFTERNOON SESSION 1

The session chaired by Jan Balvan (Czech Republic, Masaryk University) and Johannes Oesterreicher (Austria, Ludwig Boltzmann Institute for Traumatology) presented cutting-edge research on the role of EVs in various diseases, with a particular focus on cancer, regenerative medicine, and biomarker discovery. The presentations highlighted advances in proteomic analysis, isolation techniques, and therapeutic applications, underlining the growing importance of EVs in both basic research and clinical settings. Collaborative efforts within multidisciplinary networks, such as the one at Vetmeduni Vienna, further strengthen the future of EV research and pave the way for innovative diagnostic and therapeutic approaches.

The afternoon program opened with a keynote by **Alireza Fazeli** (Estonia, Tartu University), President of the Baltic Society of Extracellular Vesicles, who explored the critical role of EVs in communication between the embryo and maternal cells during the pre-implantation stage of pregnancy. He highlighted how EVs, including exosomes and microvesicles, transport biomolecules like microRNAs, facilitating key reproductive processes such as gamete maturation and implantation, with promising implications for assisted reproductive technologies. Alireza also discussed challenges in understanding how stress and cell culture systems impact EV cargo and biological effects, presenting a study comparing EVs from 2D monolayer and 3D spheroid trophoblast-like cell cultures. Notably, 3D cultures secreted more EVs with distinct proteomic profiles, while 2D-derived EVs triggered stronger responses from endometrial epithelial cells, underscoring the significance of the microenvironment. He concluded by emphasizing the importance of selecting appropriate EV production platforms for *in vitro* studies, given their impact on EV functionality and therapeutic potential.

**Helena Kupcova Skalnikova** (Czech Republic, Charles University) introduced her research group at the First Faculty of Medicine, which studies the proteome of EVs to understand the molecular mechanisms in diseases such as cancer and neurodegeneration. They have successfully characterized thousands of proteins within EVs from various sources, including patient samples and animal models, contributing to the search for new diagnostic and therapeutic targets.

**Silvio Kau-Strebinger** (Austria, Veterinary Medicine University of Vienna) presented the work of Vetmeduni Vienna, which has become a central hub for multidisciplinary EV research. Vetmeduni’s research ranges from disease-specific biomarker discovery to the development of regenerative therapies using EVs derived from MSCs. Their newly established EV-NETWORK platform promotes collaboration and innovation within the field.

**Christian Preußer** (Germany, Philipps-University Marburg) presented the results of a proteomic study of EVs derived from ovarian cancer spheroids using a 3D culture model to investigate the tumor microenvironment. While most ovarian cancer EV research has focused on ascites fluid, this study specifically examined EVs directly secreted by tumor spheroids.

**Beate Rinner** (Austria, Medical University Graz) presented the work of the Core Facility for Alternative Biomodels and Preclinical Imaging at the Medical University of Graz. Their research focuses on the use of advanced *in vitro* and *in vivo* models to study EVs released from autologous patient-derived tumor models. They employ state-of-the-art technologies such as SEC, TFF, and ExoView profiling to characterize EVs based on multiple surface markers.

In her oral presentation, **Maria Belen Arteaga** (Austria, Veterinary Medicine University of Vienna) compared the therapeutic effects of secretome fractions derived from ovine fetal MSCs on inflamed chondrocytes *in vitro*. The study found that EVs exhibited superior pro-regenerative effects by upregulating key extracellular matrix genes, highlighting their potential in therapeutic applications for tissue regeneration.

**Kerstin Menck** (Germany, University of Muenster), President of the German Society for Extracellular Vesicles, discussed the role of Syntenin in regulating adhesion proteins on small EVs and its influence on tumor progression. Her research uncovered that knocking out syntenin in breast cancer cells significantly reduced the expression of adhesion-related proteins on EVs. This affected the EVs’ capacity to bind to fibronectin and reduced their ability to promote cancer cell migration. These findings suggest that syntenin may be a target for therapeutic strategies aimed at reducing tumor metastasis.

## DAY 2 - AFTERNOON SESSION 2

The last session was chaired by Jan Pribyl (Czech Republic, Central European Institute for Technology, Masaryk University) and Michaela Stoiber (Austria, Medical University of Graz). These presentations collectively emphasized the significant role of EVs in various diseases and therapeutic applications, as presented in the oral talks. Continued research is essential to establish EVs as reliable biomarkers and therapeutic agents while addressing challenges in their analysis. These findings will provide new insights into disease mechanisms and potential treatment strategies.

The final scientific session of the conference began with a presentation by **Johannes Zipperle** (Austria, Ludwig Boltzmann Institute of Traumatology). His talk focused on the diagnostic potential of EVs in COVID-19 patients and those on extracorporeal membrane oxygenation. The study found a significant increase in erythrocyte-derived EVs in intensive care patients, suggesting shear effects during extracorporeal membrane oxygenation. These findings suggest that EVs and associated microRNAs (miRNAs) may serve as valuable biomarkers for assessing disease severity.

**Lydie Plecita-Hlavata** (Czech Republic, Czech Academy of Sciences) discussed her study of exosome cargo from pancreatic beta cells and its relation to Type 2 diabetes. By analyzing pancreatic islets from control, prediabetic, and diabetic rodents, she aimed to uncover new therapeutic strategies for managing diabetes mellitus.

In her talk, **Carla Tripisciano** (Austria, Medical University of Vienna) noted that saliva-derived EVs from individuals with severe hemophilia A contain tissue factor/factor VIIa complexes, which initiate clotting in their own factor VIII-deficient blood. This discovery may account for the low occurrence of mucosal bleeding in individuals with hemophilia A.

**Birgit Hirschmugl** (Austria, Medical University of Graz) examined placenta-derived EVs in her presentation, highlighting their altered composition in preeclampsia and its impact on inflammatory and metabolic processes. Her research aims to clarify the role of small EVs in fetal innate immunity during complicated pregnancies.

**Michaela Stoiber** (Austria, Medical University of Graz), PhD student of Birgit Hirschmugl, presented findings on umbilical cord blood CD34+ (UCB-CD34+) cells and their uptake of EVs, indicating involvement in immune communication. Her working hypothesis assesses the miRNA-induced interferon-gamma (IFN-γ) pathway’s role in hematopoietic stem cell fate during preeclampsia.

**Barbara Ukropcova** (Slovakia, Slovak Academy of Sciences) discussed how acute exercise alters circulating EV dynamics, demonstrating specific changes in molecular cargo in response to exercise in healthy young individuals. Ongoing studies will further evaluate the effects of lifestyle interventions on EVs across various populations.

**Jozef Ukropec** (Slovakia, Slovak Academy of Sciences) noted in his talk that weight loss interventions modulate EVs and their response to acute exercise in adults with obesity, suggesting their role in systemic responses to lifestyle changes.

**Małgorzata Małys** (Austria, Medical University of Vienna) presented a study titled “Small EVs are released *ex vivo* from residual leukocytes and platelets remaining in plasma”, highlighting the potential of small EVs as biomarkers for autoimmune diseases, particularly rheumatoid arthritis and anti-neutrophil cytoplasmic antibody-associated vasculitis. Her findings demonstrate that using platelet-poor plasma samples, in contrast to plasma and serum samples, minimizes complications from *ex vivo* small EV generation, enhancing the reliability of biomarker analysis.

**Karyna Tarasova** (Austria, Veterinary Medicine University of Vienna) compared the therapeutic efficacy of EVs from various sources on inflamed adult chondrocytes in her presentation, revealing that fetal MSC- and chondrocyte-derived EVs exhibit superior pro-regenerative effects compared to perinatal sources. The study highlights the importance of selecting EVs based on donor age and differentiation approach to enhance therapeutic outcomes.

## AWARDS

The conference celebrated exceptional contributions by presenting awards for the three Best Oral Presentations and three Best Posters.

At the closing ceremony of the conference, the winners of the top three oral presentations were announced: **Maximilian Haertinger** (Austria) for his presentation titled “Small extracellular vesicles derived from multipotent ASCs in peripheral nerve regeneration: jack of all trades, master of none?”, **Kerstin Menck** (Germany) for her talk on “Syntenin controls the expression of adhesion proteins on small extracellular vesicles”, and **Klara Hanelova** (Czech Republic) for her presentation, “The importance of extracellular vesicles as potential biomarkers of head and neck cancer”.

The winners of the Best Poster awards were also revealed: **Adrian Klepe** (Austria) for his poster on “EV uptake and release in a human *in vitro* blood-brain barrier co-culture model under ischemic conditions”, **Ilona Pavkova** (Czech Republic) for her work on “Extracellular vesicles released from macrophages infected with intracellular pathogenic bacterium *Francisella tularensis*”, and **Denisa Harvanova** (Slovakia) for her poster titled “Potential of extracellular vesicles in the treatment and diagnostics of osteoarthritis”.

## WORKSHOP ON FC ANALYSIS OF EVS

On September 18th, 2024, the Vienna One Vision Center hosted a workshop on the Analysis of EVs by FC. The event, organized by BCLS, provided an in-depth look into both the fundamentals and advanced techniques of EV isolation and analysis, focusing particularly on UC and FC. The workshop was divided into sessions based on participants’ skill levels and included both theoretical presentations and hands-on practical sessions.

The workshop began with a welcome address by the President of ASEV, **Wolfgang Holnthoner** (Ludwig Boltzmann Institute for Traumatology) and **Pierre Paolini** from BCLS. They introduced the agenda and highlighted the significance of precise EV analysis for research and clinical applications and the long-term cooperation between ASEV and BCLS. The participants were introduced to the structure of the workshop, which catered to both beginners and advanced attendees, with separate morning and afternoon sessions based on expertise.

In the morning UC session, designed for beginners, **Lutz Erhardt** (BCLS) introduced the basics of density gradient centrifugation for EV isolation. He covered essential steps like gradient formation, sample preparation, and how to address common issues that can affect the purity and yield of EVs. This session provided practical strategies to ensure successful isolation, offering foundational knowledge on the technique.

In contrast, the afternoon session aimed at more experienced participants delved deeper into advanced troubleshooting and optimization of density gradient centrifugation. Erhardt discussed the advantages and disadvantages of top-down *vs.* bottom-up UC approaches, with a focus on their impact on the purity of EV isolation. Special attention was given to the effects of prolonged UC times on EV yield and quality. Participants explored how minor changes in parameters, such as centrifugation speed and sample handling, could dramatically alter outcomes, with strategies provided to avoid contamination and improve yields when working with difficult biological samples.

The session dedicated to FC analysis of EVs was led by **Toni Weinhage** (BCLS) and **Maximilian Haertinger** (Medical University of Vienna). In the beginner’s part, they covered key concepts such as experiment design, controls, and the setup of single-particle detection. The participants learned how to avoid common pitfalls when handling small particles, including proper calibration, gating strategies, and experimental planning. This session emphasized the importance of precise instrument setup and control usage to ensure accurate EV detection.

For the more experienced participants in the afternoon session, Toni and Maximilian provided a deeper dive into advanced FC techniques. This session focused on small particle detection and instrument calibration, with detailed insights into optimizing laser settings, fluorescent labeling, and resolving differences in particle size. Troubleshooting challenges such as detection sensitivity and signal noise were also discussed, with practical tips to ensure precise results.

Both the morning and afternoon sessions concluded with practical, hands-on training using the CytoFLEX system. In the morning hands-on session, beginners learned how to set up the CytoFLEX for small particle detection. They practiced sample acquisition and adjusted instrument settings for the accurate detection of EVs.

The afternoon hands-on session for advanced participants built on this, offering more sophisticated training with the CytoFLEX nanosystem. Participants calibrated the instrument, fine-tuned detection limits, and acquired complex EV samples. BCLS experts were on hand to guide participants through real-time troubleshooting and optimization, ensuring they gained a comprehensive understanding of CytoFLEX’s full capabilities.

This combination of theoretical and hands-on training in both FC and UC allowed participants to enhance their practical skills, whether they were new to EV analysis or experienced researchers looking to refine their techniques.

## CONCLUSION

The ASEV-CzeSEV2024 joint meeting was a resounding success, providing a platform for scientific exchange, networking, and industry-academia collaboration. The topics covered, from basic EV biology to cutting-edge drug delivery applications, reflect the growing recognition of EVs as key players in diagnostics and therapeutics. The conference emphasized the importance of interdisciplinary collaboration and laid the groundwork for future innovations in the EV fields. Additional information can be found on the conference website (https://www.asev.at/annualmeeting).

## DECLARATIONS

### Copyright

© The Author(s) 2024.
